# The genome sequence of the Beautiful Hook-tip, *Laspeyria flexula *(Denis & Schiffermüller, 1775)

**DOI:** 10.12688/wellcomeopenres.20351.1

**Published:** 2023-11-16

**Authors:** Douglas Boyes, Mark L Blaxter, University of Oxford and Wytham Woods Genome Acquisition Lab, Darwin Tree of Life Barcoding collective, Wellcome Sanger Institute Tree of Life programme, Wellcome Sanger Institute Scientific Operations: DNA Pipelines collective, Tree of Life Core Informatics collective, Darwin Tree of Life Consortium

**Affiliations:** 1 UK Centre for Ecology & Hydrology, Wallingford, England, UK; 2 Tree of Life, Wellcome Sanger Institute, Hinxton, England, UK

**Keywords:** Laspeyria flexula, Beautiful Hook-tip, genome sequence, chromosomal, Lepidoptera

## Abstract

We present a genome assembly from an individual male *Laspeyria flexula* (the Beautiful Hook-tip; Arthropoda; Insecta; Lepidoptera; Erebidae). The genome sequence is 450.9 megabases in span. Most of the assembly is scaffolded into 31 chromosomal pseudomolecules, including the Z sex chromosome. The mitochondrial genome has also been assembled and is 15.58 kilobases in length. Gene annotation of this assembly on Ensembl identified 13,281 protein coding genes.

## Species taxonomy

Eukaryota; Metazoa; Eumetazoa; Bilateria; Protostomia; Ecdysozoa; Panarthropoda; Arthropoda; Mandibulata; Pancrustacea; Hexapoda; Insecta; Dicondylia; Pterygota; Neoptera; Endopterygota; Amphiesmenoptera; Lepidoptera; Glossata; Neolepidoptera; Heteroneura; Ditrysia; Obtectomera; Noctuoidea; Erebidae; Erebinae; *Laspeyria*; *Laspeyria flexula* (Denis & Schiffermüller, 1775) (NCBI:txid938238).

## Background

The Darwin Tree of Life project, as part of our goals of sequencing all eukaryotic species in Ireland and Britain ( Blaxter *et al.*, 2022), is generating high quality reference genomes for many lepidoptera. Here we present a chromosomally-complete genome sequence for the Beautiful Hook-tip *Laspeyria flexula* ( [Denis & Schiffermüller], 1775), a member of subfamily Boletobiinae within the noctuid family Erebidae (see NBN Atlas Partnership, 2023). Erebid moth species form approximately 4% of the Lepidoptera fauna of Britain and Ireland, including representatives of 63 genera (UK Species Inventory; Natural History Museum, 2023). Erebid moths are important herbivores, including invasive pests of native and agricultural ecosystems, but some species also act as sentinels for the impacts of anthropogenic impacts such as pesticide use and pollution and of the impacts of climate change ( Fox, 2013).

While larvae of most moths feed on vascular plant material, some, including *L. flexula*, feed on lichens. The well-camouflaged caterpillars of *L. flexula* feed on foliose and crustose lichens that grow on the bark of both deciduous and coniferous trees. Lichenivorous moths are threatened not only through generalised habitat loss but also especially through the impacts of atmospheric pollution on their food sources. It is well recognised that atmospheric pollution, most notably SO _2_, resulting from the burning of fossil fuels, led to massive declines in many lichen species in Ireland and Britain, and more widely. Following acceptance of emission controls many lichens have rebounded, and lichenivorous species such as *L. flexula* are also showing recovery (albeit against a general background of declines in moth abundances ( Fox *et al.*, 2013), especially in areas that had, historically, the highest atmospheric SO _2_ levels ( Pescott *et al.*, 2015). We hope that this genome sequence will assist in analysis of the population history and recovery of *L. flexula,* and of its adaptations to lichenivory.

## Genome sequence report

**Figure 1.**
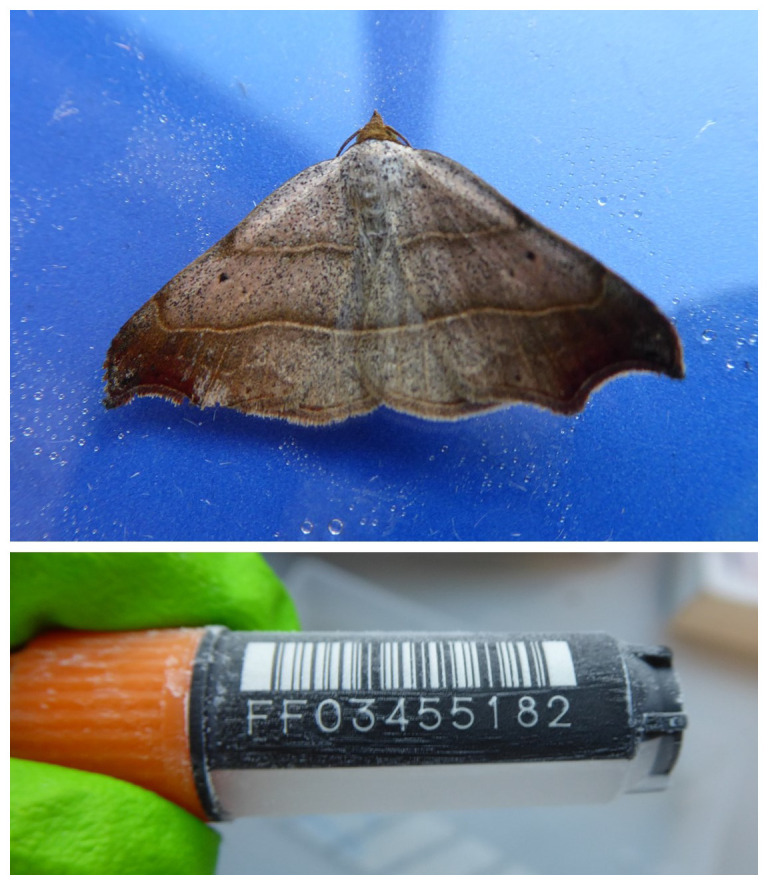
Photograph of the *Laspeyria flexula* (ilLasFlex1) specimen used for genome sequencing

The genome was sequenced from one male *Laspeyria flexula* ( Figure 1) collected from Wytham Woods, Oxfordshire, UK (51.77, –1.34). A total of 48-fold coverage in Pacific Biosciences single-molecule HiFi long reads and 84-fold coverage in 10X Genomics read clouds were generated. Primary assembly contigs were scaffolded with chromosome conformation Hi-C data. Manual assembly curation corrected 13 missing joins or mis-joins and removed 2 haplotypic duplications, reducing the assembly length by 0.23% and the scaffold number by 9.76%.

**Figure 2.**
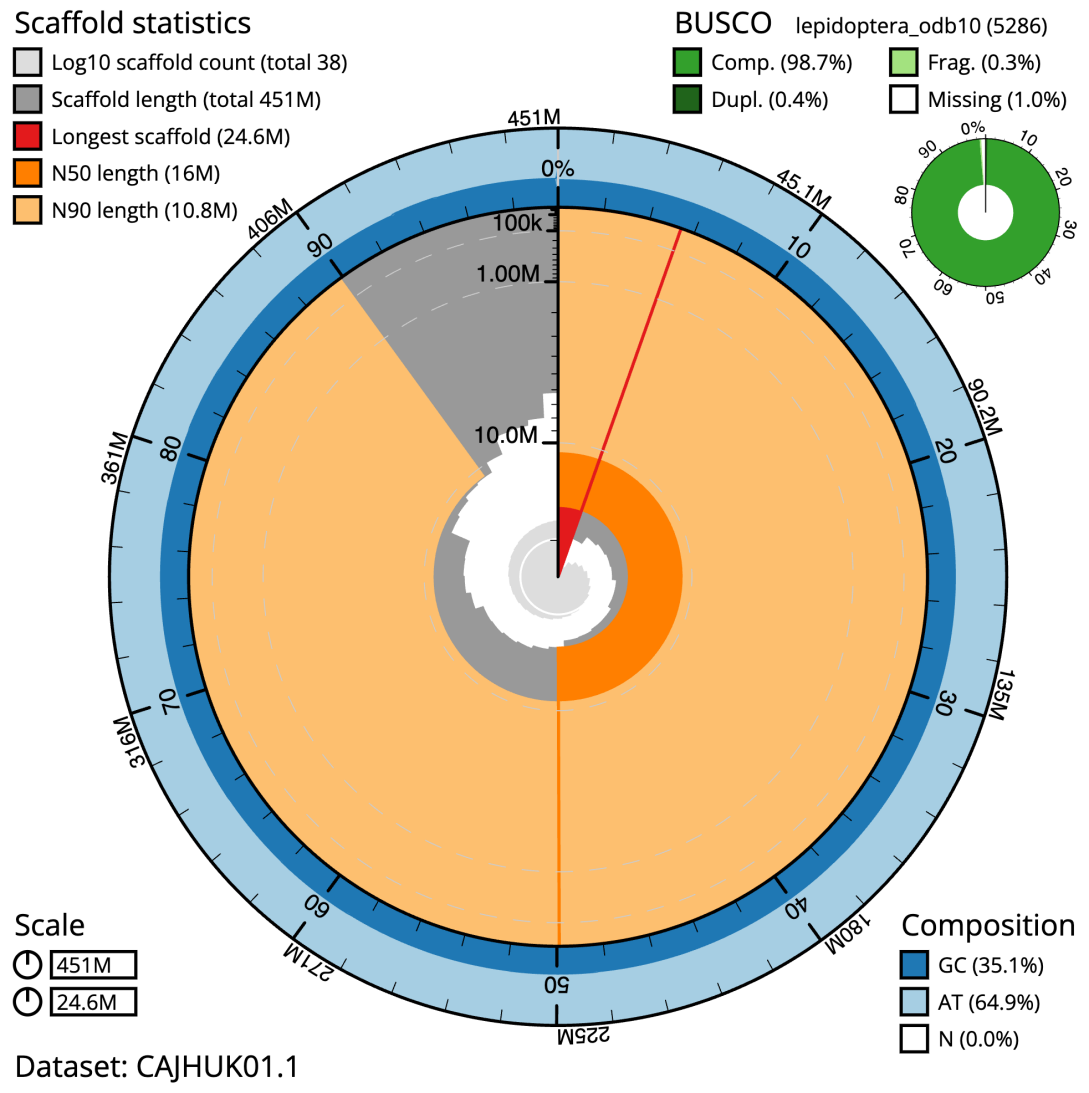
Genome assembly of *Laspeyria flexula*, ilLasFlex1.1: metrics. The BlobToolKit Snailplot shows N50 metrics and BUSCO gene completeness. The main plot is divided into 1,000 size-ordered bins around the circumference with each bin representing 0.1% of the 450,897,266 bp assembly. The distribution of scaffold lengths is shown in dark grey with the plot radius scaled to the longest scaffold present in the assembly (24,595,128 bp, shown in red). Orange and pale-orange arcs show the N50 and N90 scaffold lengths (16,004,308 and 10,809,102 bp), respectively. The pale grey spiral shows the cumulative scaffold count on a log scale with white scale lines showing successive orders of magnitude. The blue and pale-blue area around the outside of the plot shows the distribution of GC, AT and N percentages in the same bins as the inner plot. A summary of complete, fragmented, duplicated and missing BUSCO genes in the lepidoptera_odb10 set is shown in the top right. An interactive version of this figure is available at https://blobtoolkit.genomehubs.org/view/Laspeyria%20flexula/dataset/CAJHUK01.1/snail.

The final assembly has a total length of 450.9 Mb in 37 sequence scaffolds with a scaffold N50 of 16.0 Mb ( Table 1). Most (99.94%) of the assembly sequence was assigned to 31 chromosomal-level scaffolds, representing 30 autosomes and the Z sex chromosome. Chromosome-scale scaffolds confirmed by the Hi-C data are named in order of size ( Figure 2– Figure 5; Table 2). While not fully phased, the assembly deposited is of one haplotype. Contigs corresponding to the second haplotype have also been deposited. The mitochondrial genome was also assembled and can be found as a contig within the multifasta file of the genome submission.

**Table 1.** Genome data for *Laspeyria flexula*, ilLasFlex1.1.

Project accession data		
Assembly identifier	ilLasFlex1.1	
Species	*Laspeyria flexula*	
Specimen	ilLasFlex1	
NCBI taxonomy ID	938238	
BioProject	PRJEB42131	
BioSample ID	SAMEA7519836	
Isolate information	ilLasFlex1, male: whole organism (DNA sequencing and Hi-C data) ilLasFlex2: abdomen (RNA sequencing)	
Assembly metrics *		*Benchmark*
Consensus quality (QV)	58.3	*≥ 50*
*k*-mer completeness	99.9%	*≥ 95%*
BUSCO **	C:98.7%[S:98.3%,D:0.4%], F:0.3%,M:1.0%,n:5,286	*C ≥ 95%*
Percentage of assembly mapped to chromosomes	99.94%	*≥ 95%*
Sex chromosomes	Z chromosome	*localised homologous pairs*
Organelles	Mitochondrial genome assembled	*complete single alleles*
Raw data accessions		
PacificBiosciences SEQUEL II	ERR6565937	
10X Genomics Illumina	ERR6003042, ERR6002654, ERR6002655, ERR6002656	
Hi-C Illumina	ERR6002657, ERR6002658, ERR6002659	
PolyA RNA-Seq Illumina	ERR6363259, ERR6787416	
Genome assembly		
Assembly accession	GCA_905147015.1	
*Accession of alternate haplotype*	GCA_905147035.1	
Span (Mb)	450.9	
Number of contigs	57	
Contig N50 length (Mb)	12.2	
Number of scaffolds	37	
Scaffold N50 length (Mb)	16.0	
Longest scaffold (Mb)	24.6	
Genome annotation		
Number of protein-coding genes	13,281	
Number of non-coding genes	1,859	
Number of gene transcripts	23,324	

* Assembly metric benchmarks are adapted from column VGP-2020 of “Table 1: Proposed standards and metrics for defining genome assembly quality” from ( Rhie *et al.*, 2021). ** BUSCO scores based on the lepidoptera_odb10 BUSCO set using v5.3.2. C = complete [S = single copy, D = duplicated], F = fragmented, M = missing, n = number of orthologues in comparison. A full set of BUSCO scores is available at https://blobtoolkit.genomehubs.org/view/Laspeyria%20flexula/dataset/CAJHUK01.1/busco.

**Figure 3.**
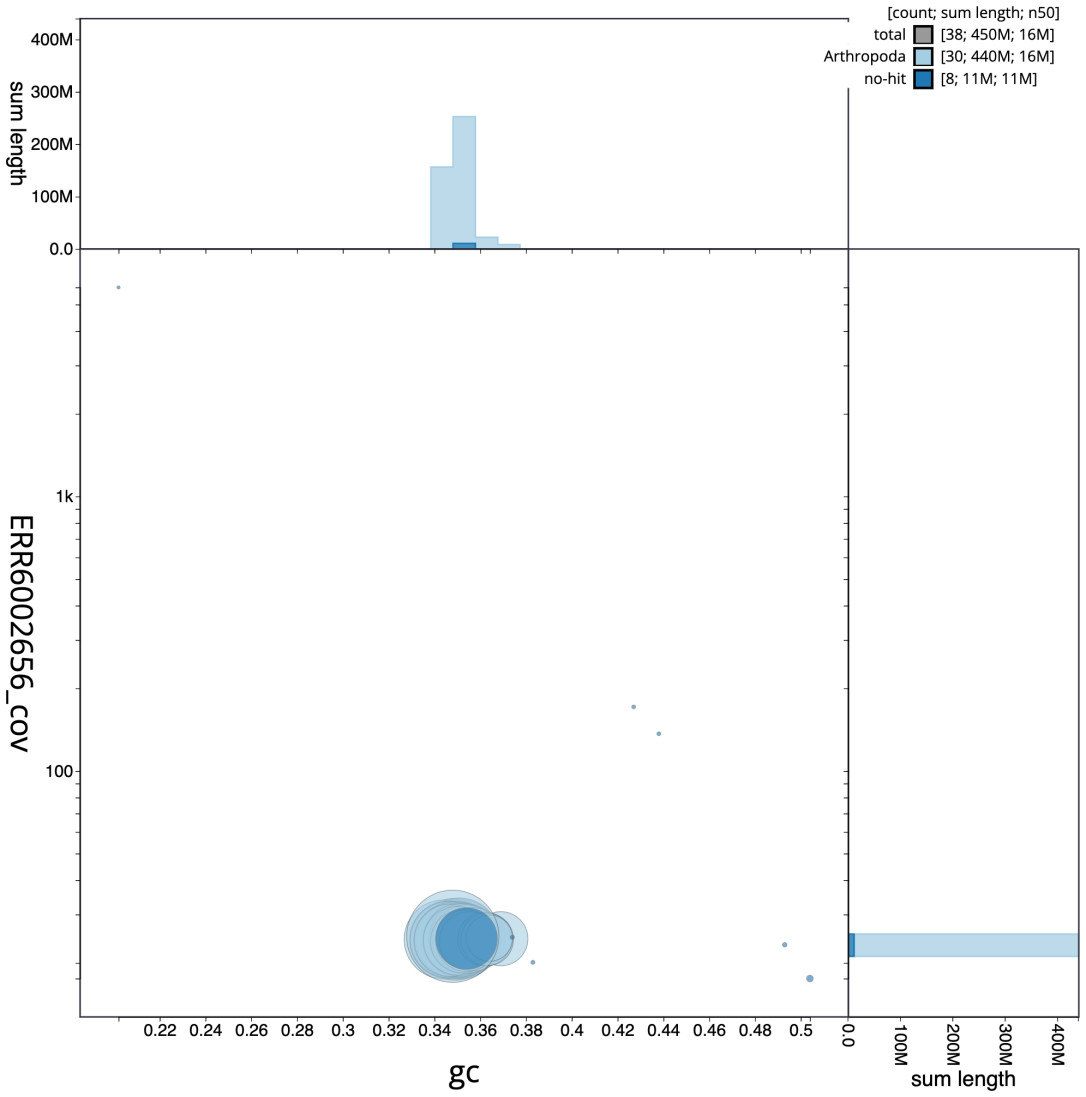
Genome assembly of *Laspeyria flexula*, ilLasFlex1.1: BlobToolKit GC-coverage plot. Scaffolds are coloured by phylum. Circles are sized in proportion to scaffold length. Histograms show the distribution of scaffold length sum along each axis. An interactive version of this figure is available at https://blobtoolkit.genomehubs.org/view/Laspeyria%20flexula/dataset/CAJHUK01.1/blob.

**Figure 4.**
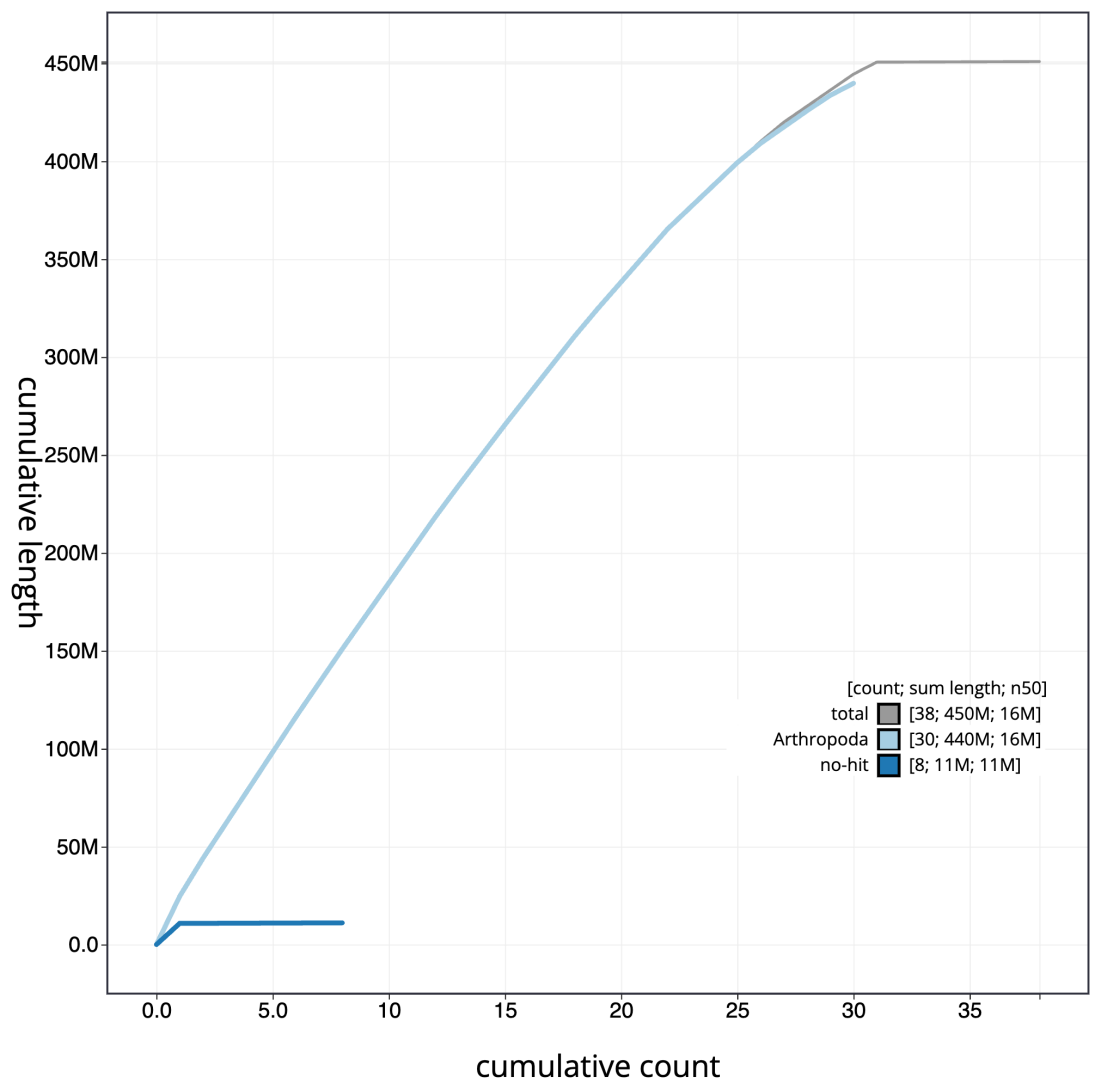
Genome assembly of *Laspeyria flexula*, ilLasFlex1.1: BlobToolKit cumulative sequence plot. The grey line shows cumulative length for all scaffolds. Coloured lines show cumulative lengths of scaffolds assigned to each phylum using the buscogenes taxrule. An interactive version of this figure is available at https://blobtoolkit.genomehubs.org/view/Laspeyria%20flexula/dataset/CAJHUK01.1/cumulative.

**Figure 5.**
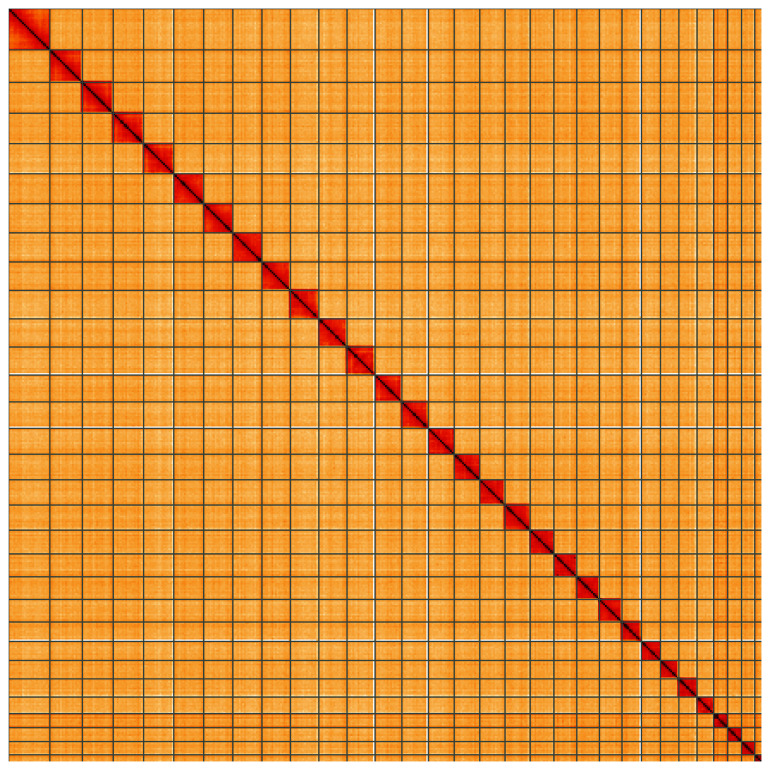
Genome assembly of *Laspeyria flexula*, ilLasFlex1.1: Hi-C contact map of the ilLasFlex1.1 assembly, visualised using HiGlass. Chromosomes are shown in order of size from left to right and top to bottom. An interactive version of this figure may be viewed at https://genome-note-higlass.tol.sanger.ac.uk/l/?d=czjwYVChQ92r5isvbpie4g.

**Table 2.** Chromosomal pseudomolecules in the genome assembly of *Laspeyria flexula*, ilLasFlex1.

INSDC accession	Chromosome	Length (Mb)	GC%
LR989950.1	1	19.38	35.0
LR989951.1	2	18.39	35.0
LR989952.1	3	18.1	35.0
LR989953.1	4	17.93	34.5
LR989954.1	5	17.83	35.0
LR989955.1	6	17.36	35.0
LR989956.1	7	17.36	35.0
LR989957.1	8	16.96	35.0
LR989958.1	9	16.93	34.5
LR989959.1	10	16.77	34.5
LR989960.1	11	16.7	34.5
LR989961.1	12	16.0	35.0
LR989962.1	13	15.74	35.0
LR989963.1	14	15.41	34.5
LR989964.1	15	15.22	35.0
LR989965.1	16	15.05	34.5
LR989966.1	17	14.97	35.0
LR989967.1	18	14.12	35.5
LR989968.1	19	13.63	35.5
LR989969.1	20	13.52	35.5
LR989970.1	21	13.45	35.0
LR989971.1	22	11.55	35.5
LR989972.1	23	11.33	35.0
LR989973.1	24	10.96	35.5
LR989974.1	25	10.81	35.5
LR989975.1	26	9.87	35.5
LR989976.1	27	8.37	37.0
LR989977.1	28	8.28	36.0
LR989978.1	29	7.87	36.5
LR989979.1	30	6.15	36.5
LR989949.1	Z	24.6	35.0
LR989980.1	MT	0.02	20.0

The estimated Quality Value (QV) of the final assembly is 58.3 with *k*-mer completeness of 99.9%, and the assembly has a BUSCO v5.3.2 completeness of 98.7% (single = 98.3%, duplicated = 0.4%), using the lepidoptera_odb10 reference set ( *n* = 5,286).

Metadata for specimens, spectral estimates, sequencing runs, contaminants and pre-curation assembly statistics can be found at https://links.tol.sanger.ac.uk/species/938238.

## Genome annotation report

The *Laspeyria flexula* genome assembly (GCA_905147015.1) was annotated using the Ensembl rapid annotation pipeline ( Table 1; https://rapid.ensembl.org/Laspeyria_flexula_GCA_905147015.1/Info/Index). The resulting annotation includes 23,324 transcribed mRNAs from 13,281 protein-coding and 1,859 non-coding genes.

## Methods

### Sample acquisition and nucleic acid extraction

A male *Laspeyria flexula* (specimen ID Ox000046, individual ilLasFlex1) was collected from Wytham Woods, Oxfordshire (biological vice-county Berkshire), UK (latitude 51.77, longitude –1.34) on 2019-06-29 using a light trap. A second specimen (ilLasFlex2, ToLID ilLasFlex2), collected from the same location on 2020-06-13, was used for RNA sequencing. Both specimens were collected and identified by Douglas Boyes (University of Oxford) and preserved on dry ice.

DNA was extracted at the Tree of Life laboratory, Wellcome Sanger Institute (WSI). The ilLasFlex1 sample was weighed and dissected on dry ice with tissue set aside for Hi-C sequencing. Tissue from the whole organism was cryogenically disrupted to a fine powder using a Covaris cryoPREP Automated Dry Pulveriser, receiving multiple impacts. High molecular weight (HMW) DNA was extracted using the Qiagen MagAttract HMW DNA extraction kit. Low molecular weight DNA was removed from a 20 ng aliquot of extracted DNA using the 0.8X AMpure XP purification kit prior to 10X Chromium sequencing; a minimum of 50 ng DNA was submitted for 10X sequencing. HMW DNA was sheared into an average fragment size of 12–20 kb in a Megaruptor 3 system with speed setting 30. Sheared DNA was purified by solid-phase reversible immobilisation using AMPure PB beads with a 1.8X ratio of beads to sample to remove the shorter fragments and concentrate the DNA sample. The concentration of the sheared and purified DNA was assessed using a Nanodrop spectrophotometer and Qubit Fluorometer and Qubit dsDNA High Sensitivity Assay kit. Fragment size distribution was evaluated by running the sample on the FemtoPulse system.

RNA was extracted from abdomen tissue of ilLasFlex2 in the Tree of Life Laboratory at the WSI using TRIzol, according to the manufacturer’s instructions. RNA was then eluted in 50 μl RNAse-free water and its concentration assessed using a Nanodrop spectrophotometer and Qubit Fluorometer using the Qubit RNA Broad-Range (BR) Assay kit. Analysis of the integrity of the RNA was done using Agilent RNA 6000 Pico Kit and Eukaryotic Total RNA assay.

Protocols developed by the Tree of Life laboratory are publicly available on protocols.io: https://dx.doi.org/10.17504/protocols.io.8epv5xxy6g1b/v1.

### Sequencing

Pacific Biosciences HiFi circular consensus and 10X Genomics read cloud DNA sequencing libraries were constructed according to the manufacturers’ instructions. Poly(A) RNA-Seq libraries were constructed using the NEB Ultra II RNA Library Prep kit. DNA and RNA sequencing was performed by the Scientific Operations core at the WSI on Pacific Biosciences SEQUEL II (HiFi), Illumina HiSeq 4000 (RNA-Seq) and HiSeq X Ten (10X) instruments. Hi-C data were also generated from remaining tissue of ilLasFlex1 using the Arima2 kit and sequenced on the HiSeq X Ten instrument.

### Genome assembly, curation and evaluation

Assembly was carried out with Hifiasm ( Cheng *et al.*, 2021) and haplotypic duplication was identified and removed with purge_dups ( Guan *et al.*, 2020). One round of polishing was performed by aligning 10X Genomics read data to the assembly with Long Ranger ALIGN, calling variants with FreeBayes ( Garrison & Marth, 2012). The assembly was then scaffolded with Hi-C data ( Rao *et al.*, 2014) using SALSA2 ( Ghurye *et al.*, 2019). The assembly was checked for contamination and corrected using the gEVAL system ( Chow *et al.*, 2016) as described previously ( Howe *et al.*, 2021). Manual curation was performed using gEVAL, HiGlass ( Kerpedjiev *et al.*, 2018) and Pretext ( Harry, 2022). The mitochondrial genome was assembled using MitoHiFi ( Uliano-Silva *et al.*, 2023), which runs MitoFinder ( Allio *et al.*, 2020) or MITOS ( Bernt *et al.*, 2013) and uses these annotations to select the final mitochondrial contig and to ensure the general quality of the sequence.

A Hi-C map for the final assembly was produced using bwa-mem2 ( Vasimuddin *et al.*, 2019) in the Cooler file format ( Abdennur & Mirny, 2020). To assess the assembly metrics, the *k*-mer completeness and QV consensus quality values were calculated in Merqury ( Rhie *et al.*, 2020). This work was done using Nextflow ( Di Tommaso *et al.*, 2017) DSL2 pipelines “sanger-tol/readmapping” ( Surana *et al.*, 2023a) and “sanger-tol/genomenote” ( Surana *et al.*, 2023b). The genome was analysed within the BlobToolKit environment ( Challis *et al.*, 2020) and BUSCO scores ( Manni *et al.*, 2021; Simão *et al.*, 2015) were calculated.

Table 3 contains a list of relevant software tool versions and sources.

**Table 3.** Software tools: versions and sources.

Software tool	Version	Source
BlobToolKit	4.0.7	https://github.com/blobtoolkit/blobtoolkit
BUSCO	5.3.2	https://gitlab.com/ezlab/busco
FreeBayes	1.3.1-17-gaa2ace8	https://github.com/freebayes/freebayes
gEVAL	N/A	https://geval.org.uk/
Hifiasm	0.12	https://github.com/chhylp123/hifiasm
HiGlass	1.11.6	https://github.com/higlass/higlass
Long Ranger ALIGN	2.2.2	https://support.10xgenomics.com/genome-exome/software/pipelines/latest/advanced/other-pipelines
Merqury	MerquryFK	https://github.com/thegenemyers/MERQURY.FK
MitoHiFi	1	https://github.com/marcelauliano/MitoHiFi
PretextView	0.2	https://github.com/wtsi-hpag/PretextView
purge_dups	1.2.3	https://github.com/dfguan/purge_dups
SALSA	2.2	https://github.com/salsa-rs/salsa
sanger-tol/ genomenote	v1.0	https://github.com/sanger-tol/genomenote
sanger-tol/ readmapping	1.1.0	https://github.com/sanger-tol/readmapping/tree/1.1.0

### Genome annotation

The Ensembl gene annotation system ( Aken *et al.*, 2016) was used to generate annotation for the *Laspeyria flexula* assembly (GCA_905147015.1). Annotation was created primarily through alignment of transcriptomic data to the genome, with gap filling via protein-to-genome alignments of a select set of proteins from UniProt ( UniProt Consortium, 2019).

### Wellcome Sanger Institute – Legal and Governance

The materials that have contributed to this genome note have been supplied by a Darwin Tree of Life Partner. The submission of materials by a Darwin Tree of Life Partner is subject to the **‘Darwin Tree of Life Project Sampling Code of Practice’**, which can be found in full on the Darwin Tree of Life website here. By agreeing with and signing up to the Sampling Code of Practice, the Darwin Tree of Life Partner agrees they will meet the legal and ethical requirements and standards set out within this document in respect of all samples acquired for, and supplied to, the Darwin Tree of Life Project. 

Further, the Wellcome Sanger Institute employs a process whereby due diligence is carried out proportionate to the nature of the materials themselves, and the circumstances under which they have been/are to be collected and provided for use. The purpose of this is to address and mitigate any potential legal and/or ethical implications of receipt and use of the materials as part of the research project, and to ensure that in doing so we align with best practice wherever possible. The overarching areas of consideration are:

•   Ethical review of provenance and sourcing of the material

•   Legality of collection, transfer and use (national and international)

Each transfer of samples is further undertaken according to a Research Collaboration Agreement or Material Transfer Agreement entered into by the Darwin Tree of Life Partner, Genome Research Limited (operating as the Wellcome Sanger Institute), and in some circumstances other Darwin Tree of Life collaborators.

## Data Availability

European Nucleotide Archive: *Laspeyria flexula*. Accession number PRJEB42131; https://identifiers.org/ena.embl/PRJEB42131 ( Wellcome Sanger Institute, 2021). The genome sequence is released openly for reuse. The *Laspeyria flexula* genome sequencing initiative is part of the Darwin Tree of Life (DToL) project. All raw sequence data and the assembly have been deposited in INSDC databases. Raw data and assembly accession identifiers are reported in Table 1.

## Author information

Members of the University of Oxford and Wytham Woods Genome Acquisition Lab are listed here: https://doi.org/10.5281/zenodo.4789928.

Members of the Darwin Tree of Life Barcoding collective are listed here: https://doi.org/10.5281/zenodo.4893703.

Members of the Wellcome Sanger Institute Tree of Life programme are listed here: https://doi.org/10.5281/zenodo.4783585.

Members of Wellcome Sanger Institute Scientific Operations: DNA Pipelines collective are listed here: https://doi.org/10.5281/zenodo.4790455.

Members of the Tree of Life Core Informatics collective are listed here: https://doi.org/10.5281/zenodo.5013541.

Members of the Darwin Tree of Life Consortium are listed here: https://doi.org/10.5281/zenodo.4783558.
