# Supplementary material for: The genome sequence of the Beautiful Hook-tip, Laspeyria flexula (Denis & Schiffermüller, 1775)
Source: Wellcome Open Res. 2023 Nov 16;8:529. [Version 1] doi: 10.12688/wellcomeopenres.20351.1 (PMC11413856; doi:10.12688/wellcomeopenres.20351.1)
Supplement: Peer review history

# Reviewer report: Reviewer response for version 1

**DOI:** 10.21956/wellcomeopenres.22536.r99419

**Published:** 2024-10-30

**Authors:** Jose Martinez

**Affiliations:** 1 Division of Plant Industry/Entomology, Florida Department of Agriculture and Consumer Services (Ringgold ID: 70124), Gainesville, Florida, USA

Boyes and Blaxter have presented a highly complete, well-assembled genome of the enigmatic noctuoid moth, *Laspeyria flexula* (the Beautiful Hook-tip; Denis & Schiffermüller, 1775), derived from sequencing a male specimen. This assembly, which boasts an exceptionally high BUSCO score, is organized into 31 chromosomal pseudomolecules, including a fully reconstructed Z chromosome, which was achieved through rigorous methodological precision, provides a highly detailed and high-quality genomic resource.

 The dataset is developed with a clear, well-structured rationale, supported by concise and comprehensible descriptions. The carefully selected protocols and methodologies ensure both precision and reproducibility. Highly standardized and enriched with detailed illustrations, the dataset is easily accessible and serves as an invaluable resource for diverse research applications.

 The authors emphasize the ecological importance of the Beautiful Hook-tip moth ( *Laspeyria flexula*), a species uniquely adapted to feed on lichens. This dietary specialization makes it especially vulnerable to environmental changes that threaten lichen populations. Habitat degradation, primarily caused by fossil fuel emissions, has severely reduced lichen abundance worldwide, putting species like the Beautiful Hook-tip at risk. This high-quality genomic resource offers valuable insights into the biology of these organisms and provides a foundation for future conservation efforts aimed at protecting them.

Are sufficient details of methods and materials provided to allow replication by others?

Yes

Is the rationale for creating the dataset(s) clearly described?

Yes

Are the datasets clearly presented in a useable and accessible format?

Yes

Are the protocols appropriate and is the work technically sound?

Yes

Reviewer Expertise:

Evolutionary Biology, Ecology, IPM, Integrative Biology, Entomology, Genomics

I confirm that I have read this submission and believe that I have an appropriate level of expertise to confirm that it is of an acceptable scientific standard.

# Reviewer report: Reviewer response for version 1

**DOI:** 10.21956/wellcomeopenres.22536.r99410

**Published:** 2024-09-18

**Authors:** Michael Hiller

**Affiliations:** 1 Senckenberg Nature Research Society, Frankfurt Am Main, Germany

A high-quality genome of the Beautiful Hook-tip is presented. The ecological importance of the species group and factors threatening the species are clearly described. PacBio HiFi, 10X and HiC sequencing data was used to assemble the genome. The resulting assembly is highly complete, measured by BUSCO and k-mer completeness, has a high base accuracy (QV 58.3) and consists of chromosome-level scaffolds.

 The assembly was annotated by the Ensembl rapid pipeline, annotating 13k coding genes.

 Minor comments and suggestions

 1) Which Hi-C protocol was used? Arima HiC or OmniC?

 2) I would like to see the contig N50 in Table 1. This could also be mentioned in the main text, as contiguity is probably more informative than the scaffold N50, given that virtually the entire assembly consists of chromosome-level scaffolds.

 3) How does the number of annotated protein-coding genes compare to related species that have a gene annotation.

 4) I would suggest to run BUSCO (or better compleasm) in annotation mode on the annotated 13k proteins to estimate how complete the annotation is.

Are sufficient details of methods and materials provided to allow replication by others?

Partly

Is the rationale for creating the dataset(s) clearly described?

Yes

Are the datasets clearly presented in a useable and accessible format?

Yes

Are the protocols appropriate and is the work technically sound?

Yes

Reviewer Expertise:

comparative genomics, assembly, annotation

I confirm that I have read this submission and believe that I have an appropriate level of expertise to confirm that it is of an acceptable scientific standard.

# Reviewer report: Reviewer response for version 1

**DOI:** 10.21956/wellcomeopenres.22536.r70283

**Published:** 2024-01-04

**Authors:** Rute R da Fonseca

**Affiliations:** 1 The Bioinformatics Centre, Department of Biology, University of Copenhagen, Copenhagen, Denmark

This a report presenting the genome assembly of and erebid moth, *Laspeyria flexula* assembled into 31 chromosomal pseudomolecules. This assembly is meant to be used in population history studies of *L. flexula,* a species that has suffered with the population decline of lichens in Ireland and Britain.

 Comments:

 1) In the abstract, the sentence describing the mitochondrial genome should be put in the end, as the number of protein coding genes refers to the nuclear genome.

 2) Information regarding the mitchondrial genome is missing from the corresponding table provided data link:

https://links.tol.sanger.ac.uk/species/938238

 3) It would be useful to have a link to the main assembly file:

https://www.ebi.ac.uk/ena/browser/view/GCA_905147015.1

Are sufficient details of methods and materials provided to allow replication by others?

Yes

Is the rationale for creating the dataset(s) clearly described?

Yes

Are the datasets clearly presented in a useable and accessible format?

Partly

Are the protocols appropriate and is the work technically sound?

Yes

Reviewer Expertise:

Genomics, bioinformatics

I confirm that I have read this submission and believe that I have an appropriate level of expertise to confirm that it is of an acceptable scientific standard.
